# Dislocation Hardening in a New Manufacturing Route of Ferritic Oxide Dispersion-Strengthened Fe-14Cr Cladding Tube

**DOI:** 10.3390/ma17051146

**Published:** 2024-03-01

**Authors:** Freddy Salliot, András Borbély, Denis Sornin, Roland Logé, Gabriel Spartacus, Hadrien Leguy, Thierry Baudin, Yann de Carlan

**Affiliations:** 1 Université Paris-Saclay, CEA, Service de Recherche en Matériaux et Procédés Avancés, 91191 Gif-sur-Yvette, Franceyann.decarlan@cea.fr (Y.d.C.); 2 Mines Saint-Etienne, Univ. Lyon, CNRS, UMR 5307 LGF, Centre SMS, F-42023 Saint-Etienne, France; borbely@emse.fr; 3 Thermomechanical Metallurgy Laboratory (LMTM)—PX Group Chair, École Polytechnique Fédérale de Lausanne (EPFL), CH-2002 Neuchâtel, Switzerland; roland.loge@epfl.ch; 4 Department of Materials Science and Engineering, KTH Royal Institute of Technology, SE-100 44 Stockholm, Sweden; spartac@kth.se; 5 Université Paris-Saclay, CNRS, Institut de Chimie Moléculaire et des Matériaux d’Orsay, 91405 Orsay, France; thierry.baudin@universite-paris-saclay.fr

**Keywords:** ODS steel, microstructure, cold rolling, dislocation density, X-ray diffraction, EBSD

## Abstract

The microstructure evolution associated with the cold forming sequence of an Fe-14Cr-1W-0.3Ti-0.3Y_2_O_3_ grade ferritic stainless steel strengthened by dispersion of nano oxides (ODS) was investigated. The material, initially hot extruded at 1100 °C and then shaped into cladding tube geometry via HPTR cold pilgering, shows a high microstructure stability that affects stress release heat treatment efficiency. Each step of the process was analyzed to better understand the microstructure stability of the material. Despite high levels of stored energy, heat treatments, up to 1350 °C, do not allow for recrystallization of the material. The Vickers hardness shows significant variations along the manufacturing steps. Thanks to a combination of EBSD and X-ray diffraction measurements, this study gives a new insight into the contribution of statistically stored dislocation (SSD) recovery on the hardness evolution during an ODS steel cold forming sequence. SSD density, close to 4.10^15^ m^−2^ after cold rolling, drops by only an order of magnitude during heat treatment, while geometrically necessary dislocation (GND) density, close to 1.10^15^ m^−2^, remains stable. Hardness decrease during heat treatments appears to be controlled only by the evolution of SSD.

## 1. Introduction

The development of the fourth generation of nuclear reactors is in progress world-wide. This generation meets the requirements of closing the lifecycle of nuclear fuels [1] while improving efficiency and safety. Thanks to substantial feedback since 1951 with the first reactor EBR-1 (Idaho), the sodium-cooled fast reactor (SFR) is the most advanced design. In this architecture, the reactor core environment is more severe than in a pressurized water reactor. Parts inside the core are subjected to neutron flux, causing more than 200 dpa, and operation in temperatures up to 650 °C [2,3].

Oxide dispersion strengthened (ODS) steels were identified in the 1960s as promising candidates for SFR cladding tubes [4], and also for the first wall in fusion reactors. Indeed, the body-centered cubic matrix provides favorable resistance for creep and swelling under irradiation, and the homogenous dispersion of nano-oxides increases the mechanical properties at high temperatures. The high chromium content in ferritic steels (>12 wt %) confers a better corrosion resistance compared to martensitic ones, but lowers the manufacturability.

Ferritic steel claddings are commonly shaped by cold working like pilgering to benefit from better geometrical accuracy [5,6,7,8,9,10,11]. Forming them is challenging since hardness higher than 400 HV1 may cause cladding cracks [7,8,9,10,11,12]. The absence of phase transformation in ferritic steel grade manufacturing requires high temperature intermediate heat treatments in order to soften and recover the material between the pilgering steps. Because of cold pilgering, grains are morphologically elongated along the rolling direction (RD). Also, the <110> lattice directions become aligned with the RD (α fiber) [13]. These microstructural and textural anisotropies influence mechanical properties, inducing a higher strength [14,15,16]. 

Microstructures of ODS grades are very sensitive to the chemical composition. Ukai et al. [14] concluded that a content of Y_2_O_3_ lower than 0.25 wt % leads to a recrystallized microstructure after specific heat treatment. This result has been confirmed by other studies [5,17] on Fe–12Cr–1.5W–0.26Ti–0.22Y_2_O_3_ cold-rolled tubes. However, the grade Fe–15Cr–2W–0.3 Y_2_O_3_ that presents a higher Y_2_O_3_ content also exhibits a recrystallized microstructure [18]. Quantitative data on dislocation densities and their evolution in microstructures have been little studied through recrystallization problematics.

This study highlights the microstructural stability of an Fe-14Cr (Fe-14Cr-1W-0.3Ti-0.3 Y_2_O_3_) ODS cladding tube during manufacturing. The investigated material is similar to 14YWT, which after extrusion at 850 °C, exhibits the ability to recrystallize as described in the standard CEA (The French Atomic Energy and Alternative Energy Commission) protocol in [8]. 

The newly proposed process route has been designed with two variations compared with the standard one: (i) a higher cumulated cross section reduction ratio than in [7] by using two successive passes and (ii) a removed stress release treatment compared to [7,8]. None of these changes, which allow for a higher stored energy, led to the recrystallization of the material.

Several microstructural indicators were analyzed, such as nano-oxides volume fraction, grain size, crystallographic texture and geometrically necessary dislocation (GND) densities from EBSD to understand hardness variation through the steps. In addition, X-ray line profile analysis (LPA) has been used to investigate the impact of stress release treatment on dislocation density. 

## 2. Materials and Methods

### 2.1. Materials and Shaping

**Figure 1:**
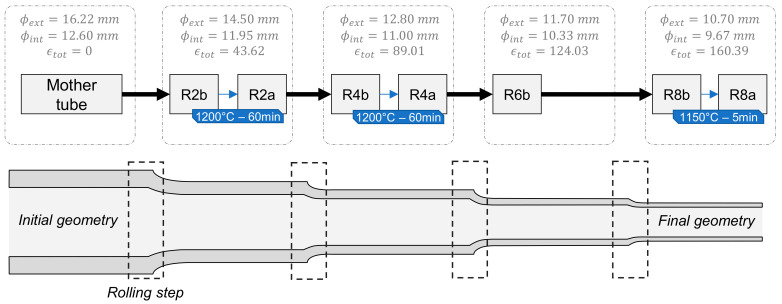
Denomination of the tube states during the rolling process.

This paper focuses on an Fe-14Cr-1W-0.3Ti-0.3Y_2_O_3_ grade steel obtained by powder metallurgy. In this process, a pre-alloyed ferritic matrix powder (Fe, Cr, W, Mn, Ni, …) atomized by Aubert & Duval was ball-milled with powders of Y_2_O_3_ and TiH_2_ by Plansee. Then, the powders were consolidated by hot extrusion. For this grade, powders and mother tube were manufactured with the same parameters as in a previous study of Toualbi et al. [7]. The processing route was designed with moderate straining by cold rolling passes (with a cumulated logarithmic reduction ratio of ~40%) and intermediate heat treatment of 60 min at 1200 °C (Figure 1). The samples were named according to the rolling pass number followed by “b” or “a”, respectively, for “before” and “after” heat treatment. Total strain $\varepsilon_{tot}$ is defined as the logarithmic ratio of the surfaces before and after rolling.

Heat treatments were performed under He atmosphere to strictly limit steel oxidation. Temperature and heating rates were monitored during heat treatment. Cooling rates were not analyzed, since they affect neither oxide precipitation nor the microstructure of ferritic steel. Ultimately, the mother tube was cold pilgered via high-precision tube rolling (HPTR) down to 500 µm wall thickness. The chemical composition is given in Table 1.

### 2.2. Experimental Techniques

All analyzed samples were taken from one cladding tube, extracted after each step of the shaping process.

**Figure 2:**
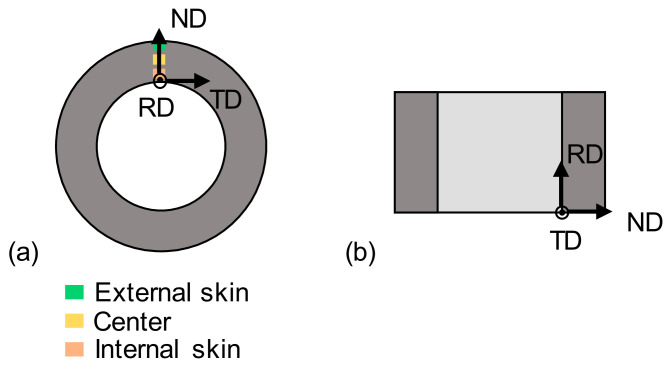
(**a**) Transverse section with the zones of interest; (**b**) longitudinal section.

Vickers hardness tests were performed using an Innovatest Falcon 500 durometer with 1 kgf load on the longitudinal [RD, normal direction (ND)] plane (Figure 2). Samples were polished until mirror surface finishing (with 1 µm diamond paste) prior to hardness testing.

Microstructural and texture characterizations were made via EBSD. Data were acquired using an SEM-FEG Zeiss SIGMA HD equipped with an Oxford fast EBSD detector, and were analyzed with the EDAX-OIM V8 software. The final preparation of EBSD samples was an electrochemical polishing that removes the hardened layer induced by diamond polishing. Orientation maps were acquired on the transverse [ND, transverse direction (TD)] plane to analyze more grains near the external skin where deformation amount is greater (Figure 2). Orientation maps were acquired with 20 kV acceleration voltage and a 35 nm step size on 21 × 28 µm^2^ area size. Texture calculations were performed using the generalized spherical harmonic series expansion approach. Calculations were performed with 34 coefficients and a 5° Gaussian half width.

Small-angle X-ray scattering (SAXS) experiments were performed on ODS steels to characterize the nano-sized oxide dispersion in the ferritic matrix. Following the same parameters as a similar study [19], data were acquired using a laboratory setup at CEA (Saclay). A Mo anode X-ray source was used (wavelength of 0.07107 nm) with a beam size of ~1 mm^2^. Samples were polished down to 80 µm thickness according to the beam energy to obtain sufficient transmission signal (10–20% of the incident intensity). The beam was aligned with ND and passed through the whole thickness of the tube.

Each SAXS measurement consisted of a two-dimensional pattern, which was azimuthally integrated, background subtracted and normalized by the incident flux, specimen thickness, transmission and solid angle viewed by the detector. Intensity was reduced to absolute units using a glassy carbon secondary standard (NIST-SRM 3600 [20]). To extract the mean radius and volume fraction, the SAXS data were fitted by a model described in [21], taking into account a lognormal distribution of spheres with a dispersion of 20% [22]. More calculation details are available in [23,24].

X-ray diffraction (XRD) patterns were acquired at the beam line P21.2 of the Petra III–DESY synchrotron (Hamburg). The X-ray energy was 83 keV, and a beam size of 150 × 150 µm^2^ was used. The beam was aligned with ND and passed through the whole thickness of the tube. A Linkam TS1500V oven heated the sample up to 1200 °C at a rate of 200 °C/min. For evaluation, the 2D XRD patterns were azimuthally integrated. The instrumental broadening was measured with a NIST LaB6 standard (SRM 660a). The modified Warren–Averbach method (mWA) was used to estimate dislocation density as described in [25]. The mWA method links the amplitude of the Fourier coefficients $A\left(n\right)$ of the line profile to the dislocation density ρ following Equation (1).

(1) $$\ln\left(A\left(n\right)\right)≅A^{S}\left(n\right)-\rho Bn^{2}\ln\left(\frac{R_{e}}{n}\right)\left(K^{2}C\right)+QB^{2}n^{4}\ln\left(\frac{R_{1}}{n}\right)\ln\left(\frac{R_{2}}{n}\right){\left(K^{2}C\right)}^{2}$$

where $A^{S}\left(n\right)$ is the small crystallite size contribution to broadening [25], *n* is the Fourier parameter, and B= $\pi b^{2}/2$ with *b* as the magnitude of the Burgers vector. $R_{e}$ is the outer cut-off radius (or screening length) of the dislocation ensemble, while $R_{1}$ and $R_{2}$ are parameters with length dimension, but no physical interpretation.

*K* = 2 sin(θ)/λ, with λ as the X-ray beam wavelength and θ as the Bragg angle. The dislocation contrast factor *C* of each diffraction peak was calculated with ANIZC using theoretical elastic constants for a ferromagnetic Fe-Cr alloy at 15 at.% Cr [26] and the assumption that edge and screw dislocations are present in equal proportion. The factor $Q=\left(\bar{\rho^{2}}-{\bar{\rho }}^{2}\;\right)/2$ represents the fluctuation of the dislocation density.

It was shown recently that the full width at half maximum (FWHM) of the peaks is strongly influenced by the arrangement of dislocations [27], and this broadening better approximates the stored energy than the value which considers $R_{e}$ evaluated from Equation (1) [28]. Its physical reason relies on the fact that Equation (1) is the asymptotic approximation of the Fourier transform of the peak profile valid at small n, which is in contradiction with the long-range interaction length between dislocations that determines their screening distance. For example, for a system of randomly arranged edge dislocation dipoles, the value of $R_{e}$ obtained from Equation (1) overestimates the true screening length by one order of magnitude [27]. Therefore, the strain energy and the corresponding screening length $R_{e}$ was evaluated using the modified Williamson–Hall method (mWH):

(2) $$\Delta K=\left(\frac{A_{D}\;}{D}\right)+{\left[\frac{b^{2}}{4\pi }\rho \ln\left(\frac{R_{e}}{r_{0}}\right)\right]}^{1/2}K\sqrt{C}+O{\left(K\sqrt{C}\right)}^{2}\;$$

where $K=2\sin\left(\theta \right)/\lambda$, $\Delta K=FWHM\cos\left(\theta \right)/\lambda$, and the inner cut-off radius $r_{0}$ was taken to be equal to 2.6 b [29].

## 3. Results and Discussion 

### 3.1. Hardness Evolution during the Manufacturing Route

**Figure 3:**
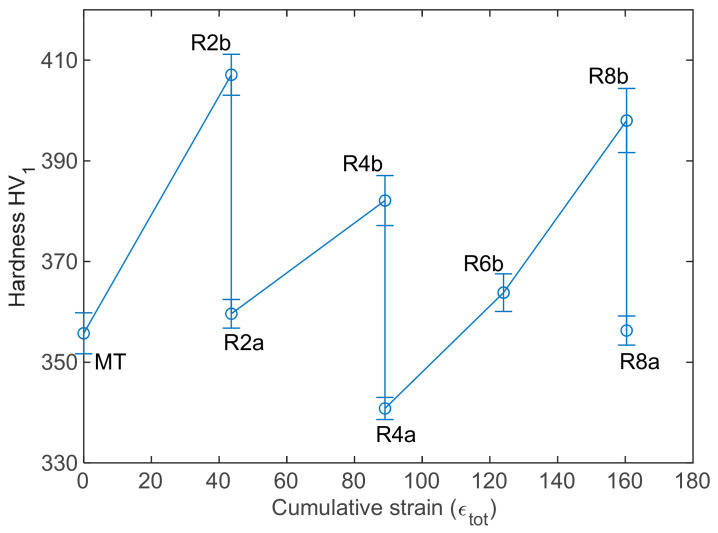
Vickers hardness at each step of the process.

The processing route was designed at CEA to reduce the mother tube (MT) to the final section by cold rolling, without damaging the tube by crack formation. The various intermediate steps are referenced in Figure 1. The total strain $\varepsilon_{tot}$ and associated hardness values are drawn in Figure 3. Obviously, each pilgering step implies hardening, while each heat treatment softens the material. 

### 3.2. Microstructural Evolution

#### 3.2.1. Nano-Oxides

SAXS has been used to measure nano-oxide size and volume fraction evolutions. Samples with α fiber texture were analyzed with the RD aligned with the X-ray beam direction to avoid an elliptic SAXS pattern related to the material texture. The assumption was made that Y_2_Ti_2_O_7_ pyrochlore was the only nano-oxides phase for the estimation of the volume fraction ($f_{V})$, as it is the most commonly identified phase in the literature [30,31]. R8* is a sample similar to R8a with a 750 °C, 30 min long heat treatment. Results are displayed in Table 2 and show a slight increase in the nano-oxide mean radius (*R_m_*) during the manufacturing from 1.4 nm to 2.0 nm. Taking into account the thermal history of consolidation and extrusion of the material to obtain the MT state, the initial radius obtained is in agreement with radius observed or calculated in [21,32] after heating up to 1100 °C. 

The growth of the nano-oxides is most likely due to coalescence during the successive thermal treatments, as the volume fraction seems to remain constant. The mean radius variations remain small, as Y_2_Ti_2_O_7_ pyrochlore is known to exhibit a strong stability against coarsening even at a high temperature [33]. Knowing that nano-oxide density N can be calculated with Equation (3), it comes to N ≈ 10^23^–10^24^ m^−2^, in agreement with previous TEM observations [31].

(3) $$N=\frac{f_{V}}{\frac{4}{3}\pi R_{m}^{\;3}}$$

This high density exerts high Zener pressure at every step of the manufacturing, and is responsible for grain boundary and dislocation pinning [34].

#### 3.2.2. Grain Size, Texture and Dislocation Density

**Figure 4:**
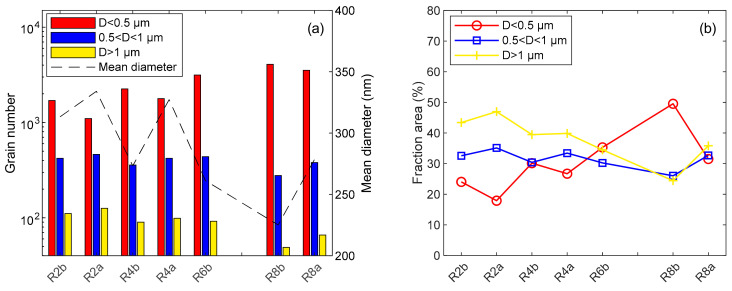
Evolution of mean diameter and grain populations in [ND, TD] section, near external skin through shaping process (**a**) in number; and (**b**) in area fraction.

**Figure 5:**
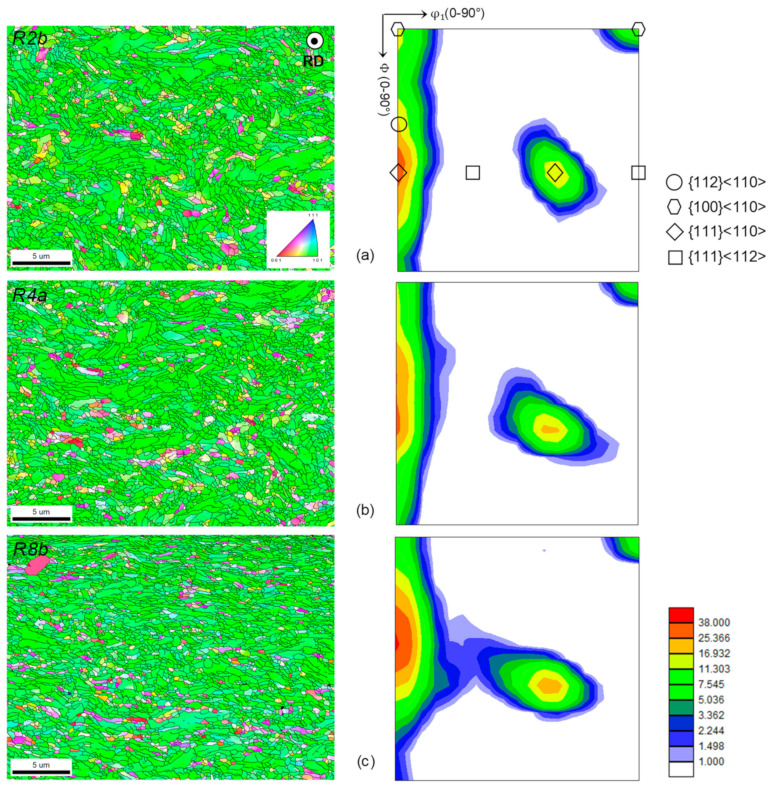
RD—IPF on TD plane on left side and Euler section at *φ*_2_ = 45° at external skin on right side for (**a**) R2b, (**b**) R4a and (**c**) R8b steps.

EBSD is a powerful method to extract microstructure and texture data from orientation maps. Analyses were performed on each step of the forming process on the [ND, TD] section to characterize their evolutions. The grain boundary was defined by considering a disorientation greater than 10°. The grain size was mostly below a 5 µm equivalent diameter (D) with a non-Gaussian distribution. So, three grain populations were studied to compare microstructures: D < 0.5 µm, D > 1 µm and the population between. Figure 4 depicts these three population variations through a shaping route. The mean equivalent diameter is also given as a comparative tool. Bigger grains (D > 1 µm) decreased in number but also in area fraction, meaning bigger grains were subdivided during the whole process. Thus, smaller grains (D < 0.5 µm) doubled in number and their area fraction increased after each cold rolling pass, impacting the mean grain size of the cladding. Microstructure tends to be more fine-grained when adding deformation cycles (Figure 5). 

Body-centered cubic (BCC) materials are well known to develop α fiber texture during rolling. The corresponding grains have crystallographic directions <110> aligned with RD [34]. This fiber is induced by extrusion [35] and is reinforced by pilgering. Regarding the outer skin, the fiber remains complete throughout the process (Figure 5) with a {111}<110> reinforcement. However, the preferential orientation tends to shift towards the {112}<110> orientation through the steps. These small and elongated (in RD) morphologies are not the most favorable for creep strength [36], but even heat treatments at 1250 °C, at whatever moment in the fabrication route, do not recrystallize the microstructure. 

The texture evolution is consistent with previous work [37,38], which evidenced the formation of a strong incomplete fiber (between {001}<110> and {111}<110>) during cold rolling of different BCC materials. Raabe and Lücke [37] report, in the case of low-carbon steels, a dominance of the fiber components {001}<110> and {112}<110>.

EBSD can also be used to calculate the geometrically necessary dislocation (GND) density [39]. Ashby [40] differentiates GNDs as dislocations tilting the crystal lattice to accommodate plastic strain and statistically stored dislocations (SSDs), which are randomly trapped dislocations that do not affect the orientation of the crystal lattice. So, only GND can be detected by conventional EBSD, and the lattice curvature is used to obtain their density $\rho_{GND}$. In this study, the Nye’s tensor has been evaluated with the first neighbor, according to the method described in [41].

**Figure 6:**
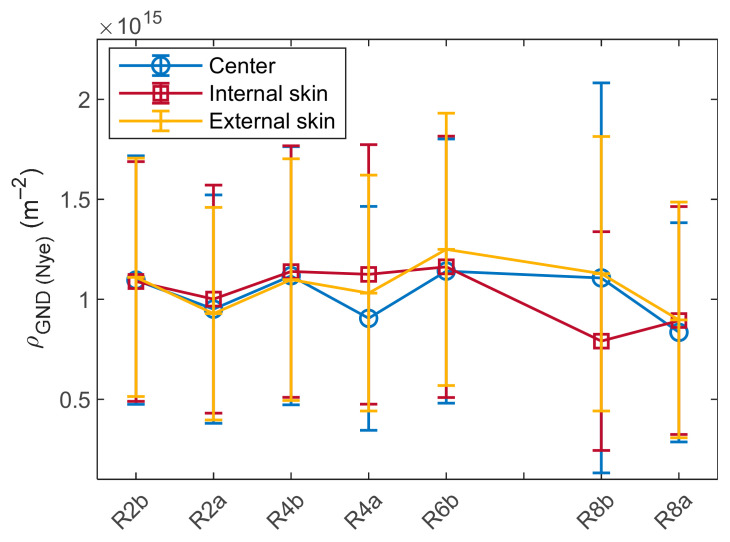
Average GND densities during the shaping process calculated by Nye’s tensor method for different sites.

The evolution of average $\rho_{GND}$ over the manufacturing process was evaluated at the center, at the internal skin and at the external skin of the tube and is presented in Figure 6. The error bars correspond to the standard deviation. $\rho_{GND}$ stays mostly constant and high (~1·10^15^ m^−2^). No clear fluctuation was found between the three sites, but low variation can be considered between the deformation and heating steps, even if those variations remain significantly lower than the error bars. 

#### 3.2.3. Hardening Contributions

Studied microstructural parameters are well known to contribute to the yield strength, and their contributions are usually added $\sigma_{YS}=\sigma_{0}+\sigma_{SS}+\sigma_{GB}+\sqrt{\sigma_{P}^{2}+\sigma_{D}^{2}}$ with $\sigma_{0}$ as the Peierls–Nabarro’s stress, $\sigma_{SS}$ as the solid solution contribution, $\sigma_{GB}$ as the contribution of grain boundaries,$\;\sigma_{P}$ as that of nanoparticles and $\sigma_{D}$ as the dislocation hardening [42,43]. According to Tabor [44], “the Vickers hardness is equal to the flow stress of a test specimen after it has been plastically strained an additional 8%”. Also, $\sigma_{YS}$ has been experimentally found to be proportional to the hardness [45], with $\sigma_{YS}$ (MPa) = hardness (MPa)/3 (1 HV_1_ = 9.81 MPa).

$\sigma_{0}$ was calculated according to the Peierls–Nabarro equation:

(4) $$\sigma_{0}=M\frac{2\mu }{1-\nu \;}\exp\left(\frac{-2\pi a}{b\left(1-\nu \right)}\right)$$

where *M* is the mean Taylor factor (*M* = 2.5 for textured material), and μ and ν are the shear modulus (81 GPa) and the Poisson’s ratio (0.3), respectively. *a* and *b* are, respectively, the lattice parameter and the modulus of the Burgers vector. $\sigma_{0}$ = 18 MPa in this study.

$\sigma_{SS}$ was estimated based on the Lacy and Gensamer relation [46] $\sigma_{SS}=\sum_{i}k_{i}X_{i}^{Z}$. $k_{i}$ and $X_{i}$ are, respectively, the atomic concentration and the hardening constant associated with each atom i. The exponent Z depends on the nature of the element in solution. It is 0.5 for insertion elements and 0.75 for larger elements in substitution. $\sigma_{SS}$ was 145 MPa according to chemical composition (Table 1).

The nano-oxides contribution $\sigma_{P}$ was estimated with the modified Orowan equation proposed by Martin [47]:

(5) $$\sigma_{P}=\frac{0.81M\mu b}{2\pi \left(1-\nu \right)2}\ln\left(\frac{1}{b}\sqrt{\frac{2}{3}}R_{m}\right)\;/\sqrt{\frac{2\pi }{3f_{V}}R_{m}}$$

Using $R_{m}$ and $f_{V}$ values from Table 2 gives $\sigma_{P}\left(MT\right)$= 350 ± 61 MPa, $\sigma_{P}\left(R2a\right)$= 304 ± 52 MPa and $\sigma_{P}\left(R8^{*}\right)$ = 257 ± 42 MPa.

Regarding the previous microstructural indicators, only (i) grain boundary strengthening and (ii) work hardening are considered in micro-hardness variations, since the solid solution hardening and $\sigma_{0}$ are not expected to change. Moreover, $\sigma_{P}$ evolution cannot lead to changes in the measured hardness. 

**Figure 7:**
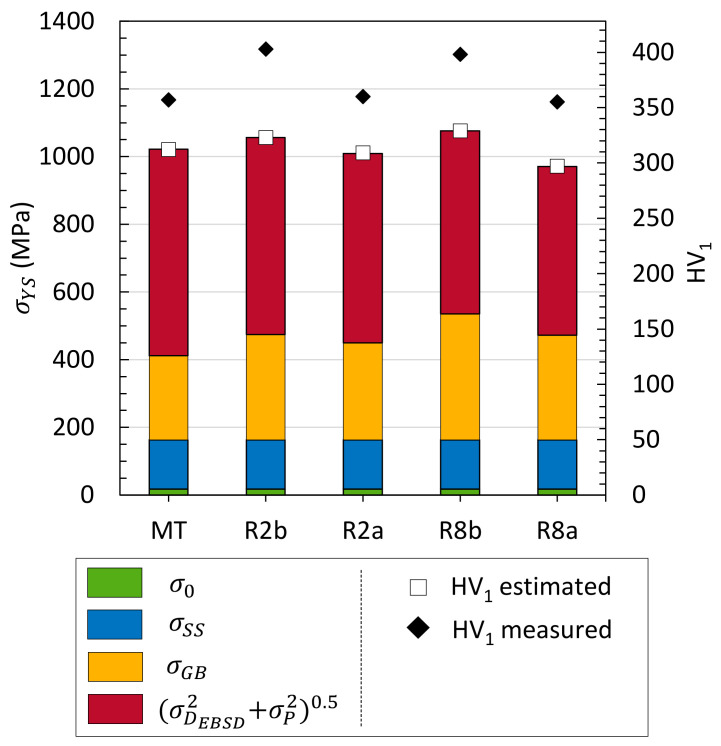
Estimated yield strength and hardness from microstructural contributions. Precipitate contribution $\sigma_{P}\left(R2b\right)$ is taken to be equal to $\sigma_{P}\left(MT\right)$ by assumption on a limited nano-oxide distribution evolution through cold pilgering step. The same for $\sigma_{P}\left(R8b\right)$, taken to be equal to $\sigma_{P}\left(R8a\right)$.

(i) Concerning the grain boundaries, the Hall–Petch effect is well known to explain the dependency of the yield stress (or hardness) with grain size [48]. To overcome the non-gaussian distribution of grain size, the Hall–Petch model modified by Srinivasarao et al. [49] was used.

(6) $$\sigma_{GB}=0.2\mu \sqrt{b}\;\underset{i}{\sum }f_{i}^{area}/\sqrt{D_{i}}\;$$

where $f_{i}^{area}$ is the area fraction of grains with a diameter *D_i_*. EBSD data were used to calculate $\sigma_{GB}$.Values are displayed in Figure 7. The grain boundary contribution to yield strength evolves similarly to hardness variations through all thermomechanical steps. $\sigma_{GB}$ increases during pilgering steps (from MT to R2b) and decreases during annealing (from R8b to R8a). However, MT and R8a show similar hardness values (Figure 3) with a higher difference between $\sigma_{GB}\left(MT\right)$ and $\sigma_{GB}\left(R8a\right)$ than between successive steps $\sigma_{GB}\left(MT\right)$ and $\sigma_{GB}\left(R2a\right)$. Hardness evolution cannot be fully explained by the Hall–Petch law.

(ii) In pure face-centered cubic (FCC) crystals, the hardness is proportional to $\sqrt{\rho }$ through the Taylor’s relation $\sigma_{D}=0.3M\mu b\sqrt{\rho }$. For BCC phases like Fe-14Cr, this relation is not as clear as for FCC [50]. The GND density assessed by EBSD does not vary significantly (Figure 6), and considering their participation alone leads to estimation close to $\sigma_{D_{GND}}$ = 470 MPa (Figure 7). Many theoretical or experimental studies have been performed on FCC materials to correlate the amount of GND and SSD densities and their respective participation in work hardening, but no consensus has been found [40,50,51,52,53].

Finally, the GND density seems to saturate; however, it shows a very slight increase through pilgering steps, and a decrease upon heat treatment (Figure 6).

By considering all these microstructural contributions, the estimated hardness values are underestimated and do not fluctuate as much as the measured ones (Figure 7). This is particularly true for variations through heat treatment (from R2b to R2a, and from R8b to R8a). At this point, none of the microstructural contributions showed any significant variation, which could explain this evolution.

#### 3.2.4. Heat Treatment Behavior

**Figure 8:**
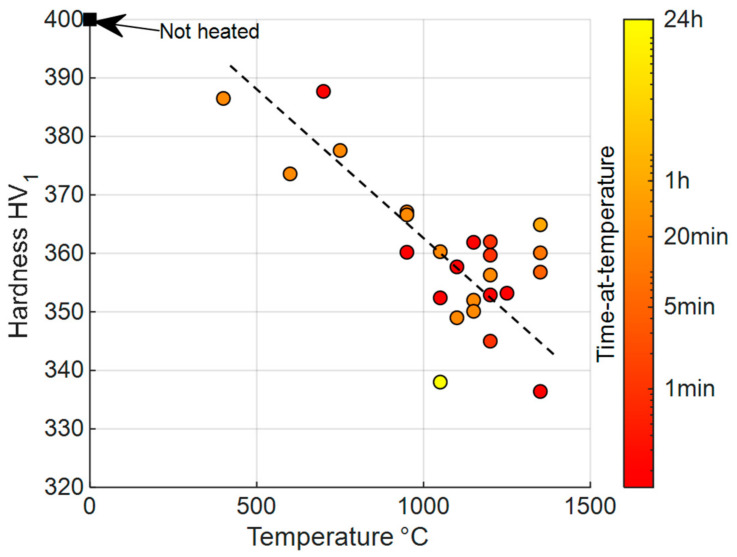
Hardness of R8b heated with various parameters.

In order to characterize the microstructure stability at various temperatures, a thorough study has been performed on the sample corresponding to the last processing step R8b. In addition, samples were maintained at the desired temperature for different durations under a helium atmosphere, followed by helium quench. As no significant evolution was noticed on the grain size distribution, hardness was used to analyze the impact of heat treatment (Figure 8). 

First, little softening is noticed until 400 °C. Then, for higher temperatures, hardness can be considered as linearly decreasing with temperature. Finally, only the temperature seems to affect hardness at first order, without any variation in the microstructure observed by EBSD. Based on this result, heat treatments lasting less than a few hours can be wisely chosen in the manufacturing route to soften cladding before pilgering (Figure 8). Moreover, since extreme treatments (fast kinetics and low holding time) impact hardness as much as moderate ones, dislocation recovery is more likely to be responsible for a hardness decrease than slower mechanisms such as chemical diffusion. Since the GND density remains stable even after 5 min at 1150 °C (Figure 6), the SSD contribution must be considered. 

**Figure 9:**
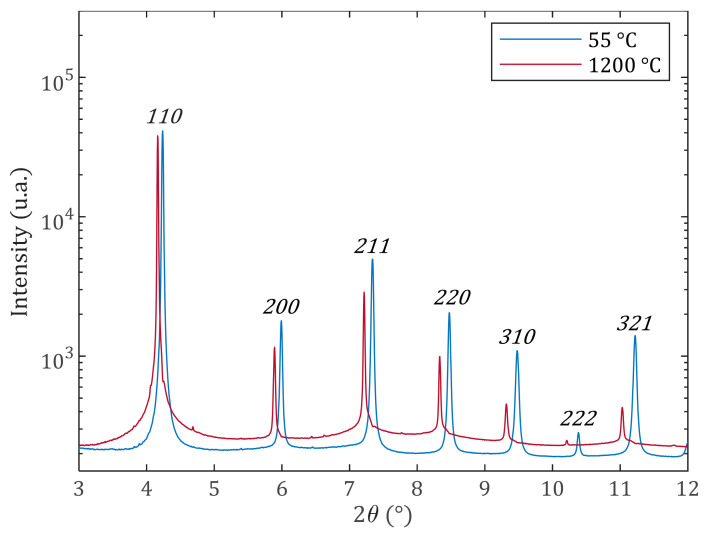
Average synchrotron X-ray diffractograms spectra measured on R8b at 55 °C and 1200 °C.

The R8b sample was analyzed by XRD with the beamline of Petra III-DESY, in order to estimate the SSD density during heating. Considering the most severe treatment possible, the sample was heated at a rate of 200 °C/min, up to 1200 °C. The acquisition of diffraction patterns occurred every second. In situ obtained average diffractograms (integrated over the azimuthal angle of the 2D detector) at 55 °C and 1200 °C are shown in Figure 9. The decrease in the intensity at higher 2θ during heating was caused by the Debye–Waller effect. Due to its low intensity, the 222 peak was not taken into account in the analysis. The 110 peak contains more than 75% of counted photons due to the strong α fiber texture. 

**Figure 10:**
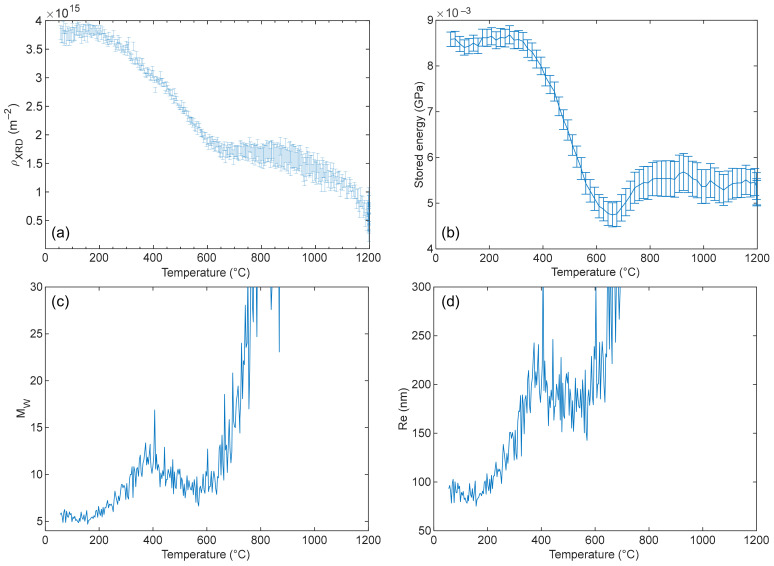
Evolution of (**a**) dislocation density, (**b**) stored energy, (**c**) the $M_{W}=R_{e}\sqrt{\rho_{XRD}}$ parameter, and (**d**) $R_{e}$ during the heating from mWA and mWH calculations.

The dislocation density from the mWA analysis dropped from 3.8 × 10^15^ ± 1.2 × 10^14^ m^−2^ to 5.1 × 10^14^ ± 1.8 × 10^14^ m^−2^ during heating (Figure 10a). Since a density near 10^13^ m^−2^ is expected in recrystallized materials, it was concluded that only recovery occurs in the sample. 

The stored energy (SE) takes into account the dislocation density but also the dislocation arrangement and can be directly obtained from the mWH plot [28]. Based on Figure 10a,b, the SE shows a similar behavior to the dislocation density. It remains stable from room temperature to about 300–400 °C, then both drop until 650 °C. Combining the SE values with the dislocation density given by the mWA method, the screening parameter $R_{e}$ and the interaction parameter $M_{W}=R_{e}\;\sqrt{\rho }$ [54] can be calculated (Figure 10d and Figure 10c, respectively).

- From room temperature to 400 °C, $\rho_{XRD}$ decreases and $M_{W}$ increases (Figure 10c). The latter describes the average dislocation [28]. The increase in both $R_{e}$ and $M_{W}$ can be understood in terms of the annihilation of dislocation dipoles for which *R_e_* is small [28]. Although both the dipoles and sub-grain boundaries have a small $M_{W}$, the latter are stable structures at high temperatures, as seen before with $\rho_{GND}$ evolution.
- From 400 to 600 °C, both $\rho_{XRD}$ and $M_{W}$ decline slightly. This means that SSD start to annihilate with a faster kinetics than bellow 400 °C. Since $M_{W}$ and $R_{e}$ are decreasing (Figure 10d), assumption can be made on the disappearance of dislocations with longer screening length. In addition, some dislocations can rearrange into GND structures (with lower energy), also leading to a decrease in $M_{W}$ and $R_{e}$.
- The 650–700 °C temperature range corresponds to the sample Curie point measured by calorimetry for this sample. Anomalous behavior in the lattice parameters [55] and also in diffusion kinetics are well known around the Curie point in ferritic stainless steels. In the studied alloy, these changes are local and since the material did not transform completely to the FCC phase (small austenite peak is visible near the 110 refection above 800 °C, Figure 9), they could induce extra strain, affecting peak broadening. As the measurements were acquired during heating, crossing the Curie point, this anomalous behavior could lead to the observed increase in $M_{W}$ and $R_{e}$, describing the dislocation rearrangement. The interpretation of these parameters above 600 °C is not possible. This austenitic phase disappears again after cooling.

Table 3 lists the dislocation density estimated from EBSD and mWA of the sample before heat treatment (R8b) and after heat treatment (R8a). Both XRD acquisitions were performed at 55 °C, and others were perfmored at room temperature. The GND density measured by EBSD was slightly reduced, while $\rho_{XRD}$ was divided by about eight. This reduction can be attributed to SSD density.

**Figure 11:**
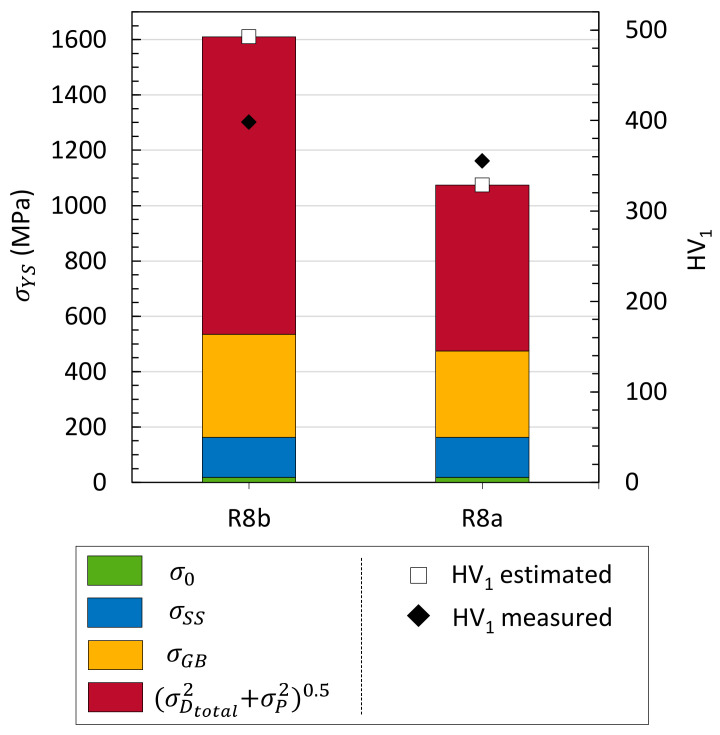
Estimated yield strength and hardness from complete microstructural contributions before and after heating up to 1200 °C at +200 °C/min. Precipitate contribution is taken constant.

Assuming that the nano-oxide distribution does not evolve during this relatively rapid heat treatment, the ${\left({\sigma_{D}^{2}}_{total}+\sigma_{P}^{2}\right)}^{0.5}$ contribution was estimated, as well as the yield strength and Vickers hardness (Figure 11).

- Hardening models

Taking $\rho_{XRD}$ into account results obviously in a greater variation in the estimated yield strength and, therefore, in the estimated hardness, than that with only GND. In the case of R8a (Figure 7 and Figure 11), the estimated hardness goes from 297 HV_1_ (EBSD) to 329 HV_1_ (EBSD + XRD) for an experimental value of 355 HV_1_. Thus, softening during annealing seems to be mainly controlled by SSDs in this highly strained and textured BCC material since the GND density does not evolve significantly.

Also, for R8b, the estimated hardness rises from 329 HV_1_ (EBSD) to 492 HV_1_ (EBSD + XRD) for an experimental value of 398 HV_1_. Then, the yield strength (and hardness) is overestimated. This can be due to several approximations.

The Taylor’s relation $\sigma_{D}=\alpha M\mu b\sqrt{\rho }$ seems not to be the right description for these materials since differences between the measured and estimated hardness values are still large. The average value of the Taylor factor M could be reconsidered (i) even though it has little impact on the estimates, as could the α-factor (ii) (here fixed at 0.3), which can vary if the dislocation structure changes [56,57]. Also, (iii) the total dislocation density may be underestimated. According to [28], the asymptotic mWA allows for a reliable evaluation of the dislocation density if the local distance between dislocations is larger than about 30 nm, which corresponds to a total dislocation density of 10^15^ m^−2^. This means that in the case of Fe-14Cr, the XRD method cannot characterize dislocation density in sub-grain and grain boundaries with disorientations larger than ~0.5°. For this reason, the mWA XRD method underestimates the total dislocation density. Finally, (iv) the Tabor’s relation remains an empirical law whose coefficient depends on the material.

- Microstructural evolution

Taking into account the evolution of the presented microstructural indicators, only the variation in SSDs and GNDs with a disorientation <0.5° seem to be responsible for the evolution of cladding hardness during the manufacturing process.

## 4. Conclusions

The Fe-14Cr-1W-0.3Ti-0.3Y_2_O_3_ grade developed at CEA exhibits high microstructure stability when being manufactured. The nano-oxide reinforcement obtained with the Y_2_O_3_ content, higher than 0.25 wt.%, limits the grain recrystallization. Stability of the microstructure persists up to 1200 °C. For an industrial shaping route, this particularity must be taken into account.

Only slight variations are noticed in all the studied microstructure indicators such as grain size, nano-oxide coalescence, texture and GND density. Hardness evolves as expected, according to heat treatments and deformation stages, while remaining around at 400 HV_1_ such as to avoid cracking. Moreover, hardness decrease is mostly controlled by the heat treatment temperature, and not by its holding time. In this way, shorter heat treatments can be envisaged.

By using a synchrotron X-ray, peak narrowing has been quantified. SSD variation in such a highly deformed BCC alloy has been identified to offer a major contribution to the measured hardness variations through thermomechanical steps. 

## Figures and Tables

**Table 1:** Chemical composition of Fe-14Cr studied grade. The carbon content is obtained by combustion with infrared detection, the nitrogen content by reductive fusion–thermal conductivity and the oxygen content by reductive fusion–infrared absorption. All other contents are measured by plasma emission spectrometry.

Content (wt.%)											
Fe	C	Cr	Mn	Mo	Ni	Si	Ti	W	Y	N	O
Bal.	0.013	14	0.25	0.005	0.33	0.28	0.27	1.1	0.15	0.015	0.13
	±0.001	±0.6	±0.02	±0.001	±0.05	±0.01	±0.01	±0.27	±0.04	±0.0005	±0.01

**Table 2:** Volume fraction and mean radius of Y_2_Ti_2_O_7_ nano-oxides calculated from SAXS signal. Standard deviation is linked to the calculated dispersion. R8* is a sample similar to R8a with a 750 °C, 30 min long heat treatment.

	MT	R2a	R8*
Volume fraction (%)	0.36 ± 0.09	0.33 ± 0.08	0.26 ± 0.07
Mean radius (nm)	1.4 ± 0.2	1.8 ± 0.2	2.0 ± 0.1

**Table 3:** Dislocation density before and after heating up to 1200 °C at +200 °C/min obtained from EBSD and XRD (calculated with the mWA method).

	R8b	R8a
$\rho_{EBSD}$ (10^15^ m^−2^)	1.0 ± 0.6	0.8 ± 0.5
$\rho_{XRD}\;$(10^15^ m^−2^)	3.8 ± 0.2	0.5 ± 0.2

## Data Availability

The data that support the findings of this study are available on request from the corresponding author Freddy Salliot.
